# Parasternal intercostal muscle thickening fraction predicts weaning failure in invasively ventilated patients with COPD: a prospective observational study

**DOI:** 10.1136/bmjresp-2026-004266

**Published:** 2026-09-11

**Authors:** Aslıhan Gürün Kaya, Deniz Doğan Mülazımoğlu, Miraç Öz Kahya, Serhat Erol, Fatma Arslan, Akın Kaya

**Affiliations:** 1 Chest Disease, Ankara University School of Medicine, Ankara, Ankara, Turkey

**Keywords:** COPD, Respiratory Muscles, Imaging/CT MRI etc, Critical Care, Pulmonary Disease, Chronic Obstructive

## Abstract

**Background:**

Weaning from invasive mechanical ventilation (IMV) is a critical phase of intensive care in patients with chronic obstructive pulmonary disease (COPD), in whom weaning failure is associated with increased morbidity and prolonged intensive care unit stay. Although diaphragm ultrasonography has been extensively studied, the contribution of accessory respiratory muscles during the weaning process remains insufficiently explored. We aimed to investigate whether parasternal intercostal muscle thickening fraction (IMTF), assessed by ultrasound, is associated with weaning outcomes in invasively ventilated patients with COPD.

**Methods:**

In this prospective observational study, patients with COPD undergoing IMV were evaluated immediately before their first spontaneous breathing trial (SBT). Parasternal intercostal muscle thickness was measured at end-expiration and end-inspiration using ultrasound, and IMTF was calculated. Weaning success or failure was defined according to standard criteria. Associations between IMTF and weaning outcomes were examined using univariable and multivariable logistic regression models adjusting for gas exchange parameters and illness severity scores.

**Results:**

A total of 131 patients were included; 106 (81.0%) were successfully weaned, while 25 (19.0%) experienced weaning failure. IMTF was significantly higher in patients with weaning failure compared with those successfully weaned (11.9±1.7% vs 7.4±1.8%, p<0.001). Higher IMTF was associated with impaired gas exchange, showing an inverse correlation with PaO₂/FiO₂ and a positive correlation with PaCO₂ measured at initiation of IMV (both p <0.001). In separate multivariable models, IMTF remained independently associated with weaning failure after adjustment for gas-exchange parameters, APACHE II score or SOFA score, with ORs ranging from approximately 3.3 to 4.0 per 1-percentage-point increase in IMTF.

**Conclusions:**

In invasively ventilated patients with COPD, increased parasternal IMTF measured immediately before the first SBT was independently associated with weaning failure. Parasternal intercostal muscle ultrasound may provide complementary information during weaning assessment.

**WHAT IS ALREADY KNOWN ON THIS TOPIC**

**WHAT THIS STUDY ADDS**

- In this prospective cohort of invasively ventilated patients with COPD, higher parasternal intercostal muscle thickening fraction measured immediately before the first spontaneous breathing trial was associated with weaning failure in separate models adjusting for gas-exchange parameters, APACHE II or SOFA.

**HOW THIS STUDY MIGHT AFFECT RESEARCH, PRACTICE OR POLICY**

- Bedside assessment of intercostal muscle activity may provide additional information when evaluating readiness for weaning in COPD and could complement diaphragm ultrasound, supporting more individualised clinical decision-making and further research in this area.

## Introduction

 Weaning from invasive mechanical ventilation (IMV) represents a vulnerable transition in intensive care, particularly for patients with chronic obstructive pulmonary disease (COPD). In this population, failure of weaning is consistently associated with prolonged ICU stay, increased morbidity and adverse clinical outcomes.^1 2^ Despite advances in ventilatory management, clinicians still lack reliable bedside markers that can identify, at the time of weaning, patients who are unlikely to tolerate the transition to spontaneous breathing.

Diaphragm ultrasonography is an established non-invasive method for assessing respiratory muscle function during liberation from mechanical ventilation. Measurements such as diaphragm excursion and diaphragm thickening fraction have demonstrated value in predicting weaning outcomes, and the feasibility and reproducibility of these measurements have been well described.^2–5^ However, obtaining reliable measurements may be difficult in some patients, particularly when increased soft-tissue thickness, abdominal distension, postoperative changes or other anatomical factors result in a suboptimal acoustic window. These factors do not usually preclude diaphragm assessment in most cases, but they may affect image quality and measurement feasibility.^6^ Additionally, in patients with COPD, chronic hyperinflation and altered chest wall mechanics may also reduce diaphragmatic excursion and complicate the interpretation of diaphragm-based measurements during weaning.^7^

When diaphragmatic contribution to ventilation is impaired, accessory respiratory muscles may be increasingly recruited to support ventilation. Parasternal intercostal muscles contribute to rib cage expansion and are increasingly recruited when ventilatory demand rises.^8–10^ In COPD, greater intercostal muscle activation has been associated with increased work of breathing and reduced ventilatory reserve, particularly during weaning attempts.^11 12^

Ultrasonographic assessment of parasternal intercostal muscle offers a technically accessible approach to evaluating changes in accessory respiratory muscle activation. Intercostal muscle thickening fraction (IMTF) has been proposed as a marker of inspiratory muscle recruitment; however, evidence supporting its prognostic role during weaning is limited. Available studies are few, heterogeneous, and rarely focused on invasively ventilated patients with COPD.^13^

Accordingly, we aimed to investigate whether parasternal IMTF measured immediately before the first spontaneous breathing trial was associated with weaning outcome in invasively ventilated patients with COPD. We hypothesised that higher IMTF would be associated with weaning failure.

To address this question, we conducted a prospective observational study in invasively ventilated patients with COPD, systematically assessing IMTF by bedside ultrasound immediately prior to the first SBT and evaluating its association with subsequent weaning outcomes.

## Methods

### Study design and setting

This prospective observational cohort study was conducted in the intensive care unit of Ankara University Faculty of Medicine, Department of Chest Diseases, between 1 October 2022 and 30 April 2025. The study protocol was approved by the local ethics committee of Ankara University (approval number: I7-390-20). Written informed consent was obtained from all patients or their legally authorised representatives prior to enrolment.

### Study population

Consecutive adult patients (≥18 years) with a confirmed diagnosis of COPD who were receiving IMV and were considered candidates for weaning were eligible for inclusion.

Patients were excluded if they had conditions that could interfere with standardised ultrasound assessment or respiratory muscle interpretation, including chest wall deformities, prior thoracic surgery affecting the parasternal region, local skin lesions preventing probe placement, known neuromuscular disease or peri-diaphragmatic pathologies influencing respiratory mechanics.

### Data collection

Baseline demographic characteristics, comorbid conditions, reason for the intensive care unit (ICU) admission, severity scores (APACHE II and SOFA), arterial blood gas values and weaning outcomes were prospectively recorded.

### Mechanical ventilation management and weaning protocol

Mechanical ventilation management and the weaning process followed a unit-specific protocol consistent with the American Thoracic Society/American College of Chest Physicians clinical practice guidelines.^14 15^

Readiness for the first SBT was assessed by the attending physician based on standardised criteria, including improvement of the underlying cause of respiratory failure, adequate level of consciousness and ability to cooperate, satisfactory gas exchange (PaO₂/FiO₂ ≥150–200 mm Hg with PEEP ≤5 cmH₂O and FiO₂ ≤0.4), haemodynamic stability with minimal or no vasopressor support and a rapid shallow breathing index ≤105 breaths/min/L.^14 15^ Arterial blood gas measurements were routinely obtained in all patients within 1 hour before the first SBT.

As part of routine practice, patients with COPD who successfully completed the first SBT were routinely extubated to prophylactic NIV unless contraindicated. During planned intervals between NIV sessions, HFNC could be used at the discretion of the treating physician, particularly to facilitate secretion clearance and provide heated, humidified respiratory support while maintaining oxygenation.

Only the first SBT attempt after fulfilment of all readiness criteria was considered for outcome assessment. Subsequent SBTs were not included in the analysis to avoid confounding by interval clinical or physiological changes.

### Intercostal muscle ultrasound assessment

Parasternal intercostal muscle thickness was assessed using bedside ultrasonography immediately before the first SBT. Ultrasound examinations were performed with a high-frequency linear transducer (HFL38, 6–13 MHz; Fujifilm Sonosite, Bothell, WA, USA) using M-mode imaging.^16^

Measurements were obtained in the sagittal plane between the second and third ribs in the parasternal region, with patients positioned at a 20° head-up angle. Intercostal muscle thickness was measured at end-expiration (Texp) and end-inspiration (Tins). IMTF was calculated as: (Tins − Texp) / Texp × 100.^8 16^ All ultrasound assessments were performed by a single experienced operator trained in lung ultrasonography. Three consecutive measurements were obtained and averaged to reduce random within-session measurement error. The parasternal intercostal muscle was identified between the second and third ribs, with the pleural line used as the deep reference for thickness measurements (see online supplemental figure S1).

### Outcomes

The primary outcome was weaning outcome following the first SBT. Weaning success was defined as successful completion of the initial SBT followed by the absence of reintubation within 48 hours after extubation. Weaning failure was defined as failure of the initial SBT or the need for reintubation within 48 hours following extubation despite initial SBT success.

### Statistical analysis

Sample size estimation was guided by previously published data from Dres *et al.*^17^ Assuming a similar difference between groups, a two-sided alpha of 0.05, 80% power and an expected weaning failure rate of approximately 20%, a total sample of around 100–120 patients, including at least 20–25 failure events, was considered sufficient to detect a clinically meaningful difference, as estimated using G*Power (version 3.1.9.6).

Continuous variables were assessed for normality using visual inspection of histograms and the Shapiro–Wilk test. Normally distributed variables are presented as mean±SD, whereas non-normally distributed variables are expressed as median with IQR. Categorical variables are reported as counts and percentages. Between-group comparisons were performed using the Student’s *t*-test or Mann–Whitney *U* test for continuous variables, as appropriate, and the χ² test or Fisher’s exact test for categorical variables. Associations between IMTF and gas-exchange parameters measured were assessed using Pearson or Spearman correlation analysis, as appropriate.

To evaluate the relationship between IMTF and weaning failure, univariable and multivariable logistic regression analyses were performed. To minimise collinearity and overfitting, separate multivariable models were constructed incorporating IMTF and established markers of respiratory impairment and disease severity, including PaO₂/FiO₂ ratio, PaCO₂, APACHE II score and SOFA score. A post hoc sensitivity model included IMTF, PaCO₂ and PaO₂/FiO₂ measured within 1 hour before the first SBT; complete pre-SBT arterial blood gas data were available for all patients.

Post hoc hierarchical analyses compared models containing APACHE II or SOFA alone with the corresponding models after addition of IMTF. Incremental model fit was assessed using likelihood-ratio χ² tests and changes in Nagelkerke R². End-expiratory and end-inspiratory thicknesses were also examined in separate exploratory univariable models, with ORs reported per 1 mm increase. Apparent model fit was described using −2 log likelihood and Nagelkerke R², without formal comparison of the non-nested models.

Results are presented as ORs with 95% CIs. A two-sided *p* value <0.05 was considered statistically significant. All statistical analyses were performed using IBM SPSS Statistics (version 22.0; IBM Corp., Armonk, NY, USA).

### Patient and public involvement

Patients and/or the public were not involved in the design, conduct, reporting or dissemination plans of this research.

## Results

During the study period, 131 invasively ventilated patients with COPD were included in the final analysis. The flow of patient inclusion and analysis is shown in online supplemental figure S2). The mean age of the study population was 73.9±8.0 years, and 64.1% were male. The most common comorbidities were hypertension (44.3%) and diabetes mellitus (19.8%). Acute exacerbation of COPD was the leading cause of ICU admission, accounting for 74.8% of cases. The median duration of IMV was 3 days (IQR 2–4), and the median length of ICU stay was 6 days (IQR 4–8). Baseline demographic characteristics, comorbidities, severity scores, and arterial blood gas parameters are summarised in table 1.

**Table 1:** Baseline demographic characteristics, comorbidities, severity scores and arterial blood gas parameters at initiation of mechanical ventilation

Characteristic	All patients (n=131)
Age, years	73.9±8.0
Male gender, n (%)	84 (64.1)
Body mass index, kg/m^2^	28.52±3.17
Comorbidities, n (%)	
Hypertension	58 (44.3)
Diabetes mellitus	26 (19.8)
Cardiovascular disease	15 (11.5)
Chronic kidney disease	19 (14.5)
Chronic liver disease	10 (7.6)
Asthma	8 (6.1)
Primary reason for ICU admission, n (%)	
Acute exacerbation of COPD	98 (74.8)
Pneumonia	45 (34.4)
Pulmonary oedema	35 (26.7)
Haemodynamic instability	13 (9.9)
Altered mental status	3 (2.3)
Severity scores, median (IQR_25-75_)	
APACHE II score	16(12–21)
SOFA score	5(4–6)
Arterial blood gases at initiation of mechanical ventilation, mean±SD	
pH	7.30±0.05
PaO₂/FiO₂	161.76±32.31
PaO₂, mmHg	64.54±13.22
PaCO₂, mmHg	62.18±8.60

Data are presented as mean±SD, median (IQR), or number (%), as appropriate. Percentages may exceed 100% as patients could have more than one comorbidity or more than one reason for ICU admission.

ABG, arterial blood gas; APACHE, Acute Physiology and Chronic Health Evaluation; COPD, chronic obstructive pulmonary disease; ICU, intensive care unit; PaCO₂, arterial carbon dioxide partial pressure; PaO₂/FiO₂, arterial oxygen partial pressure to inspired oxygen fraction ratio; pH, arterial blood pH; SOFA, Sequential Organ Failure Assessment.

### Intercostal muscle ultrasound findings

Parasternal intercostal muscle ultrasound was successfully performed in all enrolled patients. Median end-inspiratory and end-expiratory intercostal muscle thickness values were 0.58 cm (IQR 0.53–0.64) and 0.54 cm (IQR 0.48–0.59), respectively. The IMTF for the overall cohort was 8.3±2.5%. Parasternal intercostal muscle ultrasound measurements for the overall cohort are summarised in online supplemental table S1).

### Weaning outcomes

Following the first spontaneous breathing trial, 106 patients (81.0%) were successfully weaned, whereas 25 patients (19.0%) experienced weaning failure. All 25 weaning-failure events represented failure to complete the first SBT, and none of these patients underwent extubation. All 106 patients who successfully completed the first SBT were extubated to prophylactic NIV. Eleven patients (10.4%) additionally received HFNC during intervals between NIV sessions. No patient received HFNC or conventional oxygen alone, and none of the extubated patients required reintubation within 48 hours.

With respect to baseline comorbidities, cardiovascular disease was more frequent among patients who experienced weaning failure compared with those successfully weaned (24.0% vs 8.5%, p=0.040). Similarly, chronic kidney disease (32.0% vs 10.4%, p=0.011) and chronic liver disease (20.0% vs 4.7%, p=0.022) were observed more commonly in the weaning failure group. In contrast, rates of hypertension (p=0.354), diabetes mellitus (p=0.582) and asthma (p=0.648) were similar between groups.

Regarding ICU admission diagnoses, acute exacerbation of COPD was more prevalent among patients with successful weaning (80.2% vs 52.0%, p=0.003), whereas haemodynamic instability (52.0% vs 0%, p<0.001) and altered mental status (12.0% vs 0%, p=0.006) were significantly more frequent in patients who experienced weaning failure.

Patients with weaning failure had greater illness severity, with significantly higher APACHE II and SOFA scores. At initiation of IMV, they also had lower pH and PaO₂/FiO₂ ratios and higher PaCO₂ levels than patients with weaning success. Within 1 hour before the first SBT, pH and PaO₂/FiO₂ ratios were comparable between the groups, whereas PaCO₂ remained significantly higher in patients with weaning failure (table 2).

**Table 2:** Clinical characteristics and arterial blood gas parameters according to weaning outcome

Characteristic	Weaning success (n=106)	Weaning failure (n=25)	P value
Age, years	73.97±8.35	73.52±6.74	0.802
Male gender, n (%)	65 (61.3)	19 (76.0)	0.169
Body mass index, kg/m²	28.33±3.27	29.28±2.65	0.180
APACHE II score	15((11.75–17.00)	28((24.00–33.00)	<0.001
SOFA score	4((3.75–5.00)	8((5.50–9.00)	<0.001
Arterial blood gas parameters at initiation of IMV			
PaO₂/FiO₂	168.24±31.12	134.31±21.12	<0.001
pH	7.31±0.04	7.26±0.06	0.003
PaCO₂, mmHg	59.95±6.33	71.62±14.40	<0.001
Arterial blood gas parameters within 1 hour before the initiation of first SBT			
PaO₂/FiO₂	203.38±26.07	200.40±12.03	0.579
pH	7.40±0.03	7.41±0.03	0.287
PaCO₂, mmHg	51.39±5.36	56.62±5.71	<0.001

Data are presented as mean±SD, median (IQR), or number (%), as appropriate.

Weaning failure was defined as failure of the first SBT or reintubation within 48 hours after extubation. In the present cohort, all weaning failure events were attributable to failure of the first SBT.

APACHE II, Acute Physiology and Chronic Health Evaluation II; IMV, invasive mechanical ventilation; PaCO₂, arterial carbon dioxide partial pressure; PaO₂/FiO₂, arterial oxygen partial pressure to inspired oxygen fraction ratio; pH, arterial blood pH; SBT, spontaneous breathing trial; SOFA, Sequential Organ Failure Assessment.

### Association between IMTF and weaning outcome

Intercostal muscle ultrasound parameters differed markedly between patients with successful and failed weaning. Patients with weaning failure had greater intercostal muscle thickness at both end-inspiration and end-expiration, as well as a higher IMTF, than patients with weaning success (11.88±1.66% vs 7.42±1.79%, p<0.001) (table 3). In exploratory univariable analyses, end-expiratory thickness (OR 1.971, 95% CI 1.138 to 3.411; p=0.015) and end-inspiratory thickness (OR 2.628, 95% CI 1.524 to 4.532; p<0.001) were both associated with weaning outcome, while the IMTF model showed better apparent model fit in this cohort (online supplemental table S2).

**Table 3:** Parasternal intercostal muscle ultrasound parameters according to weaning outcome

Parameter	Weaning success (n=106)	Weaning failure (n=25)	P value
End-inspiratory thickness, cm	0.56 (0.52–0.62)	0.65 (0.56–0.74)	<0.001
End-expiratory thickness, cm	0.52 (0.48–0.58)	0.59 (0.49–0.66)	0.019
IMTF, %	7.42±1.79	11.88±1.66	<0.001

Data are presented as mean±SD or median (IQR), as appropriate. IMTF: intercostal muscle thickening fraction.

Weaning failure was defined as failure of the first spontaneous breathing trial or reintubation within 48 hours after extubation. In the present cohort, all weaning failure events were attributable to failure of the first spontaneous breathing trial.

**Figure 1:**
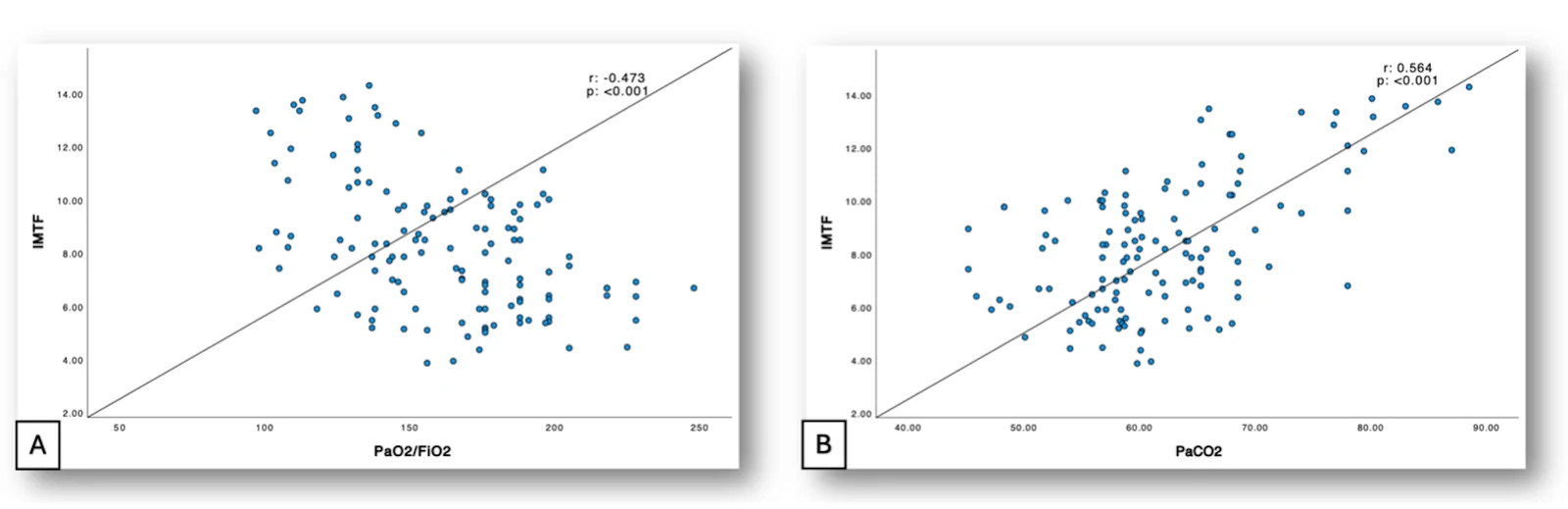
Relationship between parasternal intercostal muscle thickening fraction (IMTF) and gas exchange parameters. (**A**) Scatter plot showing the correlation between IMTF and PaO₂/FiO₂ ratio. (**B**) Scatter plot showing the correlation between IMTF and PaCO₂. Solid lines represent linear regression fits. Correlation coefficients (**r**) and p values are shown within each panel.

IMTF showed a moderate positive correlation with PaCO₂ (r=0.56, p<0.001) and a moderate negative correlation with PaO₂/FiO₂ ratio (r = −0.47, p<0.001) measured at initiation of IMV, indicating an association between higher IMTF and impaired gas exchange (figure 1).

### Multivariable analysis

In multivariable logistic regression analyses, IMTF remained independently associated with weaning failure across all models. The association persisted after adjustment for gas exchange parameters (PaO₂/FiO₂ and PaCO₂), overall illness severity as assessed by APACHE II score, and organ dysfunction as reflected by SOFA score (table 4).

**Table 4:** Multivariable logistic regression analyses of the association between IMTF and weaning outcome

	Model 1		Model 2		Model 3		Model 4	
Predictor	OR (95% CI)	p value	OR (95% CI)	p value	OR (95% CI)	p value	OR (95% CI)	p value
IMTF	3.891 (1.912 to 7.919)	<0.001	3.286 (1.339 to 8.061)	0.009	3.943 (1.916 to 8.113)	<0.001	4.043 (2.136 to 7.652)	<0.001
PaO_2_/FiO_2_ (initiation of IMV)	0.988 (0.960 to 1.016)	0.398						
PaCO_2_ (initiation of IMV)	1.014 (0.912 to 1.129)	0.791						
PaO_2_/FiO_2_ (before SBT)							1.012 (0.968 to 1.059)	0.595
PaCO_2_ (before SBT)							1.078 (0.923 to 1.259)	0.345
APACHE II			2.077 (1.182 to 3.647)	0.011				
SOFA score					2.121 (1.266 to 3.553)	0.004		
Nagelkerke R^2^	0.745		0.921		0.812		0.748	

ORs are presented with 95% confidence intervals (CIs) and correspond to a 1 percentage-point increase in IMTF, a 1 mm Hg increase in PaCO_2_, a 1-unit increase in PaO_2_/FiO_2_, and a 1-point increase in APACHE II and SOFA scores. *Model one* was adjusted for PaO_2_/FiO_2_ and PaCO_2_ at initiation of invasive mechanical ventilation. *Model two* was adjusted for APACHE II score. *Model three* was adjusted for SOFA score. *Model four* was a post hoc sensitivity analysis adjusted for PaCO_2_ and PaO_2_/FiO_2_ measured within 1 hour before initiation of the first SBT.

Weaning failure was defined as failure of the first SBT or reintubation within 48 hours after extubation. In the present cohort, all weaning failure events were attributable to failure of the first SBT.

APACHE II, Acute Physiology and Chronic Health Evaluation II; IMTF, intercostal muscle thickening fraction; IMV, invasive mechanical ventilation; PaCO₂, arterial carbon dioxide partial pressure; PaO₂/FiO₂, arterial oxygen partial pressure to inspired oxygen fraction ratio; SBT, spontaneous breathing trial; SOFA, Sequential Organ Failure Assessment.

In post hoc hierarchical analyses, adding IMTF improved model fit beyond APACHE II (likelihood-ratio χ²=17.436, df=1, p<0.001), with Nagelkerke R² increasing from 0.824 to 0.921. Adding IMTF also improved model fit beyond SOFA (χ²=42.487, df=1, p<0.001), with Nagelkerke R² increasing from 0.508 to 0.812 (online supplemental table S3).

In a post hoc analysis incorporating arterial blood gas parameters measured within 1 hour before the first SBT, IMTF remained independently associated with weaning failure (OR 4.043, 95% CI 2.136 to 7.652; p<0.001). Neither pre-SBT PaCO₂ (OR 1.078, 95% CI 0.923 to 1.259; p=0.345) nor pre-SBT PaO₂/FiO₂ (OR 1.012, 95% CI 0.968 to 1.059; p=0.595) was independently associated with the outcome (table 4).

## Discussion

In this prospective observational study of invasively ventilated patients with COPD, higher parasternal IMTF measured immediately before the first spontaneous breathing trial was associated with weaning failure. This association persisted in separate models adjusted for gas-exchange parameters, APACHE II score or SOFA score, as well as in a post hoc sensitivity analysis using pre-SBT arterial blood gas measurements. These findings suggest that parasternal IMTF may provide complementary information during weaning assessment in this population.

Successful liberation from mechanical ventilation requires an adequate balance between respiratory load and muscle capacity. Although the diaphragm is the principal inspiratory muscle, accessory respiratory muscles are increasingly recruited when diaphragmatic function is impaired or when ventilatory demand rises.^6 18^ In patients with COPD, chronic hyperinflation, altered chest wall mechanics and reduced diaphragmatic efficiency predispose to greater reliance on intercostal muscles, particularly during spontaneous breathing trials.^2 19–21^ In this setting, increased IMTF may be consistent with greater parasternal intercostal muscle recruitment during the SBT. However, because direct measures of inspiratory effort, neural respiratory drive or work of breathing were not obtained, the physiological mechanism underlying this association cannot be established from the present data.

Beyond respiratory mechanics, systemic comorbidities and the primary reason for ICU admission appear to play an important role in shaping the weaning trajectory in this population. Cardiovascular disease, chronic kidney disease and chronic liver disease were more prevalent among patients who experienced weaning failure, suggesting that a higher systemic disease burden and reduced physiological reserve may limit the ability to tolerate spontaneous breathing.^1 22^ Likewise, patients admitted with non-respiratory organ dysfunction, such as haemodynamic instability or altered mental status, demonstrated poorer weaning outcomes compared with those admitted primarily for acute respiratory decompensation.^18 22^ These observations underscore that weaning failure in COPD is not solely a respiratory phenomenon, but rather reflects the interplay between respiratory load and systemic vulnerability.^23^

Diaphragm ultrasonography has been extensively evaluated as a predictor of weaning outcomes; however, its routine applicability may be limited by technical constraints and physiological factors, including suboptimal acoustic windows and altered diaphragmatic geometry.^4 6^ Our findings extend the concept of respiratory muscle imaging beyond the diaphragm and highlight the potential value of intercostal muscle assessment as a complementary approach. In this context, parasternal intercostal muscle ultrasound may provide a non-invasive estimate of changes in accessory respiratory muscle activation during weaning.^8^ Previous surface electromyography studies in patients with stable hypercapnic COPD have also shown that intercostal muscle activity varies with the level of ventilatory assistance.^24^ However, ultrasound-derived IMTF should not be interpreted as a direct measure of inspiratory effort or work of breathing.

Evidence regarding the role of intercostal muscle ultrasound in the weaning process remains limited. Previous studies have suggested associations between parasternal intercostal muscle activity and inspiratory effort or extubation outcomes in heterogeneous ICU populations.^9 11 24 25^ By focusing on invasively ventilated patients with COPD and standardising ultrasound assessment immediately before the first spontaneous breathing trial, the prospective assessment of IMTF immediately before the first SBT allowed its association with subsequent weaning outcome to be evaluated. Absolute end-inspiratory and end-expiratory thicknesses were also associated with weaning outcome; however, the better apparent fit of the IMTF model in exploratory analyses does not establish its superiority.

From a clinical perspective, IMTF may serve as a practical bedside marker to identify patients at increased risk of weaning failure. Also, in post hoc hierarchical analyses, adding IMTF to models containing APACHE II or SOFA alone improved apparent model fit within the study sample. Intercostal muscle ultrasound is technically accessible, can be performed rapidly at the bedside, and does not rely on subcostal acoustic windows.^8 23^ Incorporation of IMTF assessment into the weaning evaluation may therefore enhance risk stratification and support clinical decision-making, particularly in patients with concomitant systemic comorbidities or limited feasibility of diaphragm ultrasonography.^6 18^

Several limitations should be acknowledged. First, this was a single-centre study, and all ultrasound examinations were performed by one experienced operator; formal intraobserver, interoperator or interoccasion reproducibility was not assessed. Although three consecutive measurements were averaged, the small absolute differences in muscle thickness warrant validation by multiple operators in larger, multicentre cohorts. Second, diaphragm ultrasound was not included in the original prospective protocol and was not performed systematically. This is an important limitation because we could not directly compare IMTF with established diaphragm-based indices, assess whether IMTF provides incremental information beyond diaphragm ultrasound or evaluate the combined value of these respiratory muscle measurements. Direct measures of inspiratory effort, neural respiratory drive and work of breathing were also unavailable, limiting mechanistic interpretation. Third, all failure events occurred during the first SBT, and all extubated patients received prophylactic NIV; therefore, the findings primarily concern initial SBT tolerance, the effect of NIV on post-extubation outcomes could not be assessed, and generalisability to centres using different support strategies may be limited. Finally, the limited number of failure events may have resulted in optimistic estimates of model performance, and causal inference is not possible because of the observational study design.

In invasively ventilated patients with COPD, increased parasternal IMTF measured immediately before the first spontaneous breathing trial was independently associated with weaning failure. These findings support further evaluation of IMTF as a complementary ultrasound parameter in weaning assessment.

## Supplementary material

10.1136/bmjresp-2026-004266 online supplemental file 1

## Data Availability

Data are available upon reasonable request.
