# Supplementary material for: Parasternal intercostal muscle thickening fraction predicts weaning failure in invasively ventilated patients with COPD: a prospective observational study
Source: BMJ Open Respir Res. 2026 Sep 11;13(1):e004266. doi: 10.1136/bmjresp-2026-004266 (PMC13583736; doi:10.1136/bmjresp-2026-004266)
Supplement: Supplementary file 1 [file bmjresp-13-1-s001.docx]

**Online Supplementary material**

**Supplementary Figure S1.** Ultrasonographic assessment of the parasternal intercostal muscle

Representative parasternal ultrasound image demonstrating the intercostal muscle between the second and third ribs. The intercostal muscle is visualized between the subcutaneous tissue and the pleural line, which serves as the deep anatomical reference for thickness measurements highlighted in yellow, with the pleural line indicated in blue.

**Supplementary Figure S2.** Flow diagram of patient selection

**Supplementary Table S1.** Ultrasonographic assessment of parasternal intercostal muscles

| **Parameter** | **All patients**  **(n = 131)** |
| --- | --- |
| End-inspiratory thickness, cm | 0.58 [0.53–0.64] |
| End-expiratory thickness, cm | 0.54 [0.48–0.59] |
| IMTF, % | 8.27 ± 2.49 |
| Data are presented as median [interquartile range] or mean ± standard deviation, as appropriate. IMTF: intercostal muscle thickening fraction | |

**Supplementary Table S2.** Exploratory univariable logistic regression analyses of parasternal intercostal muscle ultrasound parameters in relation to weaning failure

| **Predictor** | **OR (95% CI)** | **p value** | **-2 log likelihood** | **Nagelkerke R²** |
| --- | --- | --- | --- | --- |
| End-expiratory thickness, mm | 1.971 (1.138–3.411) | 0.015 | 121.570 | 0.074 |
| End-inspiratory thickness, mm | 2.628 (1.524–4.532) | <0.001 | 113.670 | 0.163 |
| IMTF, % | 4.452 (2.342–8.464) | <0.001 | 46.942 | 0.739 |
| Odds ratios are reported per 1-mm increase in end-expiratory and end-inspiratory thickness and per 1-percentage-point increase in IMTF. Lower −2 log likelihood and higher Nagelkerke R² values indicate better apparent model fit within the study sample. These analyses were exploratory and univariable, and no formal statistical comparison between the non-nested models was performed. IMTF, intercostal muscle thickening fraction. | | | | |

**Supplementary Table S3.** Improvement in model fit after addition of IMTF to APACHE II and SOFA scores

| **Model** | **Nagelkerke R²** | **Likelihood-ratio χ² for addition of IMTF** | **p value** |
| --- | --- | --- | --- |
| APACHE II alone | 0.824 | — | — |
| APACHE II + IMTF | 0.921 | 17.436 | <0.001 |
| SOFA alone | 0.508 | — | — |
| SOFA + IMTF | 0.812 | 42.487 | <0.001 |
| Likelihood-ratio χ² values compare each illness-severity score model with the corresponding model after addition of IMTF. Nagelkerke R² is a pseudo-R² measure and should not be interpreted as the proportion of outcome variance explained.  APACHE II:Acute Physiology and Chronic Health Evaluation II; IMTF, intercostal muscle thickening fraction; SOFA, Sequential Organ Failure Assessment. | | | |
